# Nucleus accumbens dynamics in food reward seeking and regulation by sleep

**DOI:** 10.1038/s41398-025-03442-z

**Published:** 2025-07-01

**Authors:** Ana L. Almeida Rojo, Li Cai, Tyler R. Barnhardt, Yanhua H. Huang

**Affiliations:** Department of Psychiatry, University of Pittsburgh, Pittsburgh, PA 15219 USA

**Keywords:** Neuroscience, Learning and memory

## Abstract

Reward-seeking behavior is essential for survival and is greatly influenced by experience, internal states, and physiological factors such as sleep. The nucleus accumbens (NAc) is reward processing hub that integrates external and internal signals to regulate reward-seeking behaviors. However, it is not well understood how NAc activities during reward seeking may be shaped by learning experience, and to what extent that it may be subject to physiological regulations such as sleep. Here, we used in vivo fiber photometry to monitor calcium (Ca^2+^) activities in the NAc of male and female mice undergoing sucrose self-administration (SA) training. We found that the NAc Ca^2+^ dynamics during sucrose SA were related to the behavioral outcome and evolved over different training stages. Moreover, acute sleep deprivation increased sucrose SA while reduced NAc Ca^2+^ responses and dampened its sensitivity to reward update. Thus, our findings suggest that the NAc response during natural reward seeking is dynamic, adaptive to learning experience, and can be blunted by acute sleep deprivation.

## Introduction

Reward seeking is essential to our survival, and reward seeking behaviors are highly adaptive. A multitude of factors regulate reward-seeking behaviors, including internal state, experience and memory, environmental cues, circadian rhythm, and sleep etc. [1, 2]. How these factors regulate the brain reward circuitry is key to our understanding of the adaptive nature of reward seeking.

The nucleus accumbens (NAc) is an interface between limbic and motor regions, in which emotional and motivational signals are integrated to facilitate motor activities [3–5]. The NAc plays an essential role in reward evaluation and processing [6–9]. In support of this, in vivo studies have revealed NAc activity dynamics in humans and animals during reward anticipation, evaluation, and/or responding [10–23]. Moreover, certain aspects of the NAc responses are dependent on reward-associated learning process [24, 25], reflecting an adaptive nature. However, it is not known how stable these reward-associated NAc responses are after initial formation, and how they may be subject to physiological regulations such as sleep.

Sleep disruptions impact reward seeking in both humans and animals [26, 27]. The underlying neural mechanisms are not well understood, though the NAc is thought to be involved. Following acute sleep deprivation (SD), human imaging studies have demonstrated greater NAc responses to reward-associated cues and/or reward deliveries, including food reward and cues [28, 29], monetary gains and risk-related decision-making [30–32]. To better understand how NAc activities may be regulated by sleep and corresponding changes in reward-seeking behaviors, we used a mouse sucrose self-administration model, and applied in vivo fiber photometry to examine real-time NAc activity dynamics during sucrose reward seeking – first through different learning stages, then tested the impact of acute SD.

## Methods

### Animals

Male and female C57BL/6 J mice (Jackson Laboratory, stock #000664), 9–15 weeks old, were randomly chosen for all experiments. Mice were maintained at room temperature (22 ± 1 °C), controlled humidity (60 ± 5%), and on a 12:12 light/dark cycle with lights on at 7:00 AM (zeitgeber time, ZT 0). Mice were singly housed once they started behavioral training. All mice had free access to food and water. Mouse usage was in accordance with protocols approved by the Institutional Animal Care and Use Committees at the University of Pittsburgh (#24024571).

### Surgery

Viral and optic fiber surgery was conducted around postnatal day (PD) 49–63. Mice were anesthetized with ketamine (100 mg/kg, IP) and xylazine (10 mg/kg, IP) and placed on a stereotaxic apparatus (Kopf Instruments). A 34-gauge injection needle connected to a Hamilton syringe driven by a microinfusion pump (Harvard Apparatus) was used to unilaterally inject 0.3–0.5 μl/site (0.1 μl/min) of the calcium indicator jRCaMP1b [33] (Addgene viral prep # 100851-AAV9) into the NAc shell (AP + 1.5, ML ± 0.70, DV −5.00). Subsequently, optic fibers (200-μm core) with flat tips were unilaterally implanted above the NAc (AP + 1.35, ML ± 0.70, DV −4.30). All mice were singly housed after surgery. Mice began behavioral training approximately 2 weeks after surgery.

### Sucrose self-administration (SA) training

All SA training was conducted in operant-conditioning chambers (Med Associates). Active-lever pressing resulted in the delivery of a single sucrose pellet (20 mg; Bio-Serv, catalog #F05301, chocolate flavored). Inactive lever pressing had no consequences but was counted. Mice underwent overnight training at fixed-ratio (FR) 3 without a fiber optic cable attached for 2 nights, then with an optic fiber cable attached for 1 one night, followed by daily 30 min training at FR1 for ~2 weeks before sleep manipulations. The mice continued with daily training after sleep manipulations till ~ 5 weeks when they completed reversal SA training. All tests were performed under FR1. A single ALP resulted in a 20-sec timeout during which both levers were withdrawn. One pellet was delivered 2 s after each ALP. The mice had *ad libitum* access to water but not standard chow during overnight training. Daily sucrose SA training was conducted at approximately ZT6. This time was chosen to match the testing time after sleep deprivation procedure (see below).

### In vivo fiber photometry

The mice were adapted to handling for 2–3 days prior to attachment to low-autofluorescence patch cords (Doric Lensens). The patch cords were photobleached for ~1 hr prior to recordings per Doric Lenses recommendation to ensure less than ~15% photobleaching during 30 min recordings. JRCaMP1b recordings started at approximately ZT6 during sucrose SA. JRCaMP1b signal in the NAc was obtained by using 560 nm light to monitor Ca^2+^ responses and 405 nm light (LEDs, Doric Lenses) as isosbestic control signal minimally dependent on Ca^2+^ activity [33, 34]. Light intensities were ~20–30 μW at the end of the patch cord and kept consistent throughout the recordings. Light was delivered into the NAc via a 200 μm 0.57 N.A. optical fiber (RWD Life Science) fitted with 1.25 mm bronze sleeve (Doric Lenses) for attachment to the optic fiber implant. A rotary joint (Prizmatix) was attached at the top of the optical fiber to maintain optimal light transmission as the mice were freely moving. JRCaMP (F_560_) and isosbestic signals (F_405_) were collected by the same optical fiber and passed through a fluorescence mini cube for filtering (FMC6_IE(400-410)_E1(460-490)_F1(500-540)_E2(555-570)_F2(580-680)_S, Doric Lenses). The signals were then focused onto a femtowatt photoreceiver (Model 2151, Newport), amplified, and A-D converted using the RZ5P processor (Tucker David Technologies). Active lever presses (ALP) and inactive lever presses (ILP) were TTL time-stamped in Synapse (Tucker David Technologies).

### Fiber photometry data analysis

Raw photometry data (F_405_, F_560_) were analyzed blind to treatment conditions using MATLAB R2022a custom-written scripts. Detrending and normalization of each ALP and ILP was calculated by dF = F_560_ / Median_560_ – F_405_ / Median_405_. dF data was downsampled to 250 Hz and cut to 30 s segments with the midpoint (t = 0) aligned to the onset of ALP TTL time stamps. dF data for each individual 30-sec segment was Z-scored using individual baselines at ~ −15 to −10 s prior to the corresponding ALP. The Z-scored data was averaged per training session per animal and used for group analyses for area-under-the-curve (AUC) and kinetics.

### Sleep deprivation

After obtaining a daily performance baseline, mice underwent regular sleep or SD for 4 h ~(ZT2–6). Mice had *ad libitum* access to food and water throughout the entirety of the SD procedure. Mice were sleep deprived through the gentle-handling method [35]. Briefly, mice were kept awake by introducing novel objects, nesting material, gentle tapping and/or moving the cage, a 1-time bedding change ~ ZT 3, and occasionally, gentle brushing of the tail and vibrissae with a soft brush. This method used in our hands introduces minimal stress to the mice as measured by plasma corticosterone levels [36]. Mice were given 3 days to 1 week to recover between tests.

### Immunohistochemistry

Mice were anesthetized with overdosed isoflurane, and then perfused transcardially with 0.1 M phosphate buffer (PB) followed by 4% paraformaldehyde in PB. Brains were removed and given an additional overnight postfix in 4% paraformaldehyde at 4 °C, and then transferred to 30% sucrose in PB for 48 h before sectioning. Coronal sections (35–40 μm) were cut on a cryostat (Leica CM1950) and were collected for imaging. Endogenous fluorescence was captured by the Olympus SLIDEVIEW VS200 slide scanner, and the virus injection sites and levels of expressions were determined based on the images.

### Statistics

Group sizes were determined based on power analyses using preliminary estimates of variance by achieving 80% power to observe differences at *α* = 0.05. Normal distribution was assumed for all data. Behavioral and fiber photometry data were analyzed in GraphPad Prism 9. One-way or two-way ANOVA (including mixed-effects analysis) was conducted followed by *post-hoc* tests. In all graphs, means were reported as mean ± SEM and *p* values were from main effects or interactions ^*^p < 0.05, ^**^p < 0.01, ^***^p < 0.001, and post-hoc tests ^#^*p* < 0.05, ^##^*p* < 0.01, ^###^*p* < 0.001, ^####^*p* < 0.0001.

## Results

### NAc Ca^2+^ activity during sucrose reward seeking is related to behavioral outcome and sensitive to training stages

**Fig. 1:**
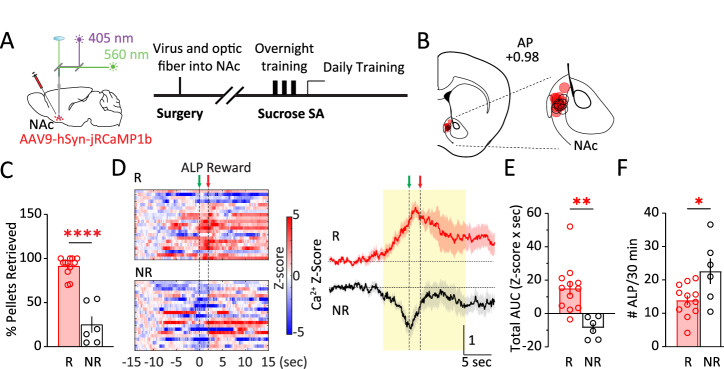
NAc Ca^2+^ activity during sucrose reward seeking is related to behavioral outcome. **A** Surgery, fiber photometry configuration, and experimental timeline. **B** Diagram of a coronal section containing the NAc, showing the positions of fiber optic tips in Retrievers (*red*) and Non-retrievers (*black*). **C** % Pellets retrieved in the two groups of mice: Retrievers (R) and Non-retrievers (NR). **D** *Left*, Representative heat maps of NAc Ca^2+^ Z-scores from a Retriever (R) versus a Non-retriever (NR) mouse during SA training over 30 min. Each row represented an individual trial with ALP aligned at t = 0 s. *Right*, Group-averaged Z-scores of NAc Ca^2+^ activity from Retriever mice versus Non-retriever mice, shown as Mean +/− SEM from all mice in each group. *Yellow shades*, periods used for AUC calculations. *Green arrow*, time of ALP; *red arrow*, time of sucrose pellet reward delivery. **E** The Retrievers had greater total Ca^2+^ activity than the Non-retrievers, calculated as AUC from −5 s till 10 s. **F** The Non-retrievers had greater #ALP than the Retrievers during the test as in **D**, **E**. ALP active-lever press, AUC area-under-curve, NAc nucleus accumbens, NR non-retriever, R retriever, SA self-administration. Data were represented as mean ± SEM. Each circle represents a mouse. n = 6 – 12 mice in each group. * *p* < 0.05, ** *p* < 0.01, **** *p* < 0.0001.

Male and female mice received unilateral intra-NAc injection of AAV expressing a genetically encoded Ca^2+^ sensor jRCaMP1b [33] and fiber optic implantation, and were trained to press an active lever to obtain sucrose pellet reward (Fig. 1A, B; Methods). After overnight trainings, most mice (12 out of 18) performed ALP and regularly retrieved the sucrose pellets (% Pellets retrieved: 91.4 ± 3.1%; Retrievers) during subsequent tests, whereas some (6 out of 18) learned to ALP but consistently retrieved less (% Pellets retrieved: 25.1 ± 9.2%; Non-retrievers; *t*_16_ = 8.572, *p* < 0.0001; *t*-test; Fig. 1C), even though the pellets were freely accessible. NAc population Ca^2+^ activities were monitored via in vivo fiber photometry and aligned to active lever-press (ALP). Whereas the Retrievers typically showed an overall increase in NAc population Ca^2+^ activities surrounding ALP and reward delivery, the Non-retrievers showed an overall decrease in NAc Ca^2+^ responses (total AUC −5 to 10 s, *t*_16_ = 3.795, *p* < 0.01; Fig. 1D, E), with a contrasting higher #ALP (*t*_16_ = 2.665, *p* < 0.05; Fig. 1F). Thus, the NAc Ca^2+^ dynamics were divergently associated with subsequent behaviors. Furthermore, the Retrievers were able to maintain a stable self-administration (SA) performance and continued with the remainder of the experiments, whereas the initial Non-retrievers gradually stopped lever-pressing for sucrose in subsequent training days and thus were not included for further experimentation. Together, these results suggest that the NAc Ca^2+^ response during reward seeking is related to the reinforcing behavioral outcome.

**Fig. 2:**
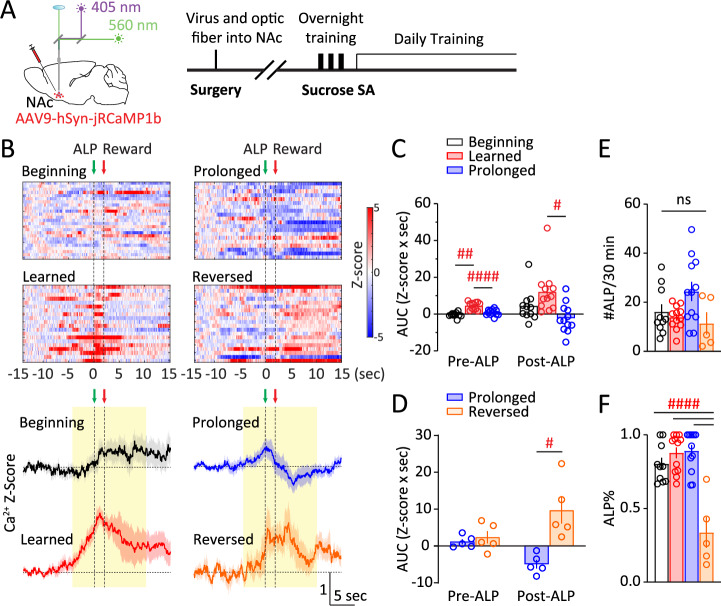
NAc Ca^2+^ activity during sucrose reward seeking is sensitive to training stages. **A** Surgery, fiber photometry configuration, and experimental timeline. **B** *Top*, Representative heat maps of NAc Ca^2+^ Z-scores from a mouse through the four stages. Each row represented an individual trial with ALP aligned at t = 0 s. *Bottom*, Averaged NAc Ca^2+^ Z-scores in all the mice across sucrose SA training stages, shown as Mean +/− SEM. *Yellow shades*, periods used for AUC calculations. *Green arrow*, time of ALP; *red arrow*, time of sucrose pellet reward delivery. **C** AUC of NAc Ca^2+^ Z-score integrated at pre-ALP (−5–0 s) or post-ALP (0–10 s) across stages i to iii. **D** AUC of NAc Ca^2+^ Z-score integrated at pre-ALP (−5–0 s) or post-ALP (0–10 s) across stages iii to iv. **E** No changes in #ALP across training stages. **F** No changes in ALP% at stages i to iii, and a decrease during stage iv. Data were represented as mean ± SEM. Each circle represents a mouse. n = 10 – 12 mice for stages i to iii, and n = 5 for stage iv. ALP active-lever press, AUC area-under-curve, NAc nucleus accumbens, SA self-administration. *Post-hoc* test # *p* < 0.05, ## *p* < 0.01, #### *p* < 0.0001; ns = not significant.

Over the following period of ~5 weeks (Fig. 2A), the NAc Ca^2+^ responses kept evolving, which were compared and contrasted under four stages (Fig. 2B): i) When mice first acquired sucrose SA (defined as #ALP/#total lever press >2/3), which typically occurred upon finishing the overnight training; ii) when #ALP stabilized over daily training, typically one week after stage i; iii) about 3–4 weeks following stage ii with stable behavioral performances; iv) in a subset of mice, the active-lever and inactive-lever were subsequently reversed. Dividing the NAc Ca^2+^ responses into two phases, pre-ALP (−5–0 s) and post-ALP (0–10 s), we found that acquisition of sucrose SA increased the pre-ALP Ca^2+^ responses (stage x phase: *F*_(1.807, 14.45)_ = 4.799, *p* < 0.05; stage i versus ii, *p* < 0.01; 2-way RM ANOVA with Tukey’s test), whereas prolonged training reduced both pre-ALP and post-ALP Ca^2+^ response (stage ii versus iii, pre-ALP: *p* < 0.0001; post-ALP: *p* < 0.05; 2-way RM ANOVA with Tukey’s test; Fig. 2B, C). The reduction in stage iii NAc Ca^2+^ responses was not because of a loss of jRCaMP1b expression or function – as we tested in a subset of mice, reversal of the active- versus inactive-levers during subsequent SA training revealed prominent Ca^2+^ activities post-ALP when the mouse pressed the newly assigned active-lever (*F*_(1, 4)_ = 17.52, *p* < 0.05; post-ALP in stage iii versus iv, *p* < 0.05; 2-way RM ANOVA with Šídák’s test; Fig. 2B, D, S1A). At the behavioral level, there was no overall difference in #ALP or ALP% across training stages, except for ALP% in reversal learning (#ALP: *F*_(3, 35)_ = 3.130, *p* < 0.05 but no significant multiple comparisons, one-way ANOVA with Tukey’s test, Fig. 2E; ALP%: *F*_(3, 35)_ = 19.27, *p* < 0.0001; reversal training compared to onset, learned, and prolonged training, *p* < 0.0001, one-way ANOVA with Tukey’s test, Fig. 2F), suggesting that the changes in NAc Ca^2+^ responses were not because of overall ALP performance differences across the training stages. Together, these results suggest that the NAc is engaged upon initial reward learning as well as during reversal learning, but less activated after prolonged training even if the performance of reward seeking is at similar levels. Moreover, the NAc is engaged from before the reward-seeking action till after reward delivery, and both phases of the NAc responses are dampened after prolonged training. We thus focused on mice during the stable responding stage ii for sleep manipulation experiments.

### Acute sleep deprivation reduces NAc Ca^2+^ activity during sucrose reward seeking

**Fig. 3:**
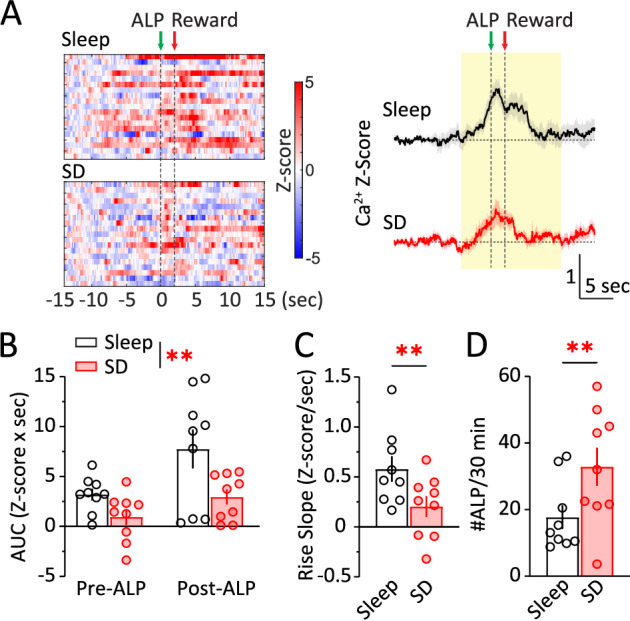
Acute sleep deprivation reduces NAc Ca^2+^ activity during sucrose reward seeking. **A** *Left*, Representative heat maps of NAc Ca^2+^ Z-scores from a mouse performing sucrose SA following normal sleep or acute SD. Each row represented an individual trial with ALP aligned at t = 0 s. *Right*, Averaged NAc Ca^2+^ Z-scores from all the mice during sucrose SA following normal sleep or acute SD, shown as Mean +/− SEM. *Yellow shades*, periods used for AUC calculations. *Green arrow*, time of ALP; *red arrow*, time of sucrose pellet reward delivery. **B** AUC of NAc Ca^2+^ Z-score integrated at pre-ALP (−5–0 s) or post-ALP (0–10 s) following normal sleep or acute SD. **C** SD reduced the rise slope of NAc Ca^2+^ responses (−2–0 s). **D** SD increased #ALP per 30 min. ALP active lever press, AUC area-under-curve, SD sleep deprivation. Data were represented as mean ± SEM. Each circle represents a mouse. n = 9 mice. **p < 0.01.

A single episode of acute sleep deprivation (SD) directly impacts reward seeking in both animals and humans [28, 29, 36–55] and impairs NAc synaptic transmission [36, 54]. How may acute SD alter NAc activities during reward seeking? We measured NAc Ca^2+^ dynamics during sucrose SA following acute SD (Fig. 3A). Compared to normal sleep days, NAc Ca^2+^ responses were decreased following acute SD (SD x phase: *F*_(1, 8)_ = 0.966, *p* = 0.355; main effect of SD, *p* < 0.01, RM 2-way ANOVA; Fig. 3B). Moreover, the rise slope of NAc Ca^2+^ at −2 – 0 s before ALP was reduced after SD (*t*_8_ = 2.975, *p* < 0.01; paired *t*-test; Fig. 3C). The decrease in NAc Ca^2+^ was accompanied by an increase in #ALP for sucrose following SD (*t*_8_ = 3.179, *p* < 0.01; paired *t*-test; Fig. 3D), and an increase in pellet consumption (*t*_8_ = 2.864, *p* < 0.05; paired t-test), consistent with previous reports that acute SD increases sucrose reward seeking in male mice [36, 54]. Together, these results suggest that acute SD reduces NAc activities while increasing sucrose reward seeking.

### Acute SD dampens NAc Ca^2+^ responses to reward update

**Fig. 4:**
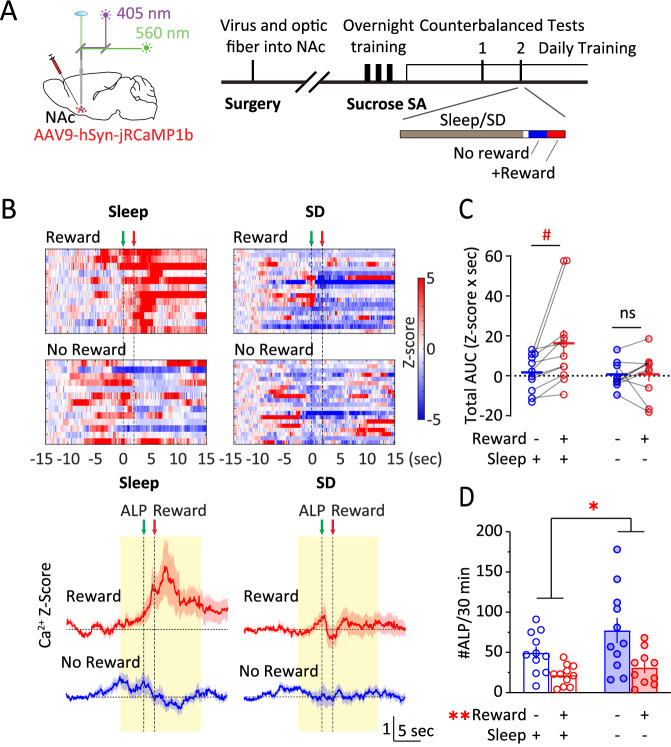
Acute sleep deprivation reduces NAc Ca^2+^ responses to reward update. **A** Surgery, fiber photometry configuration, and experimental timeline. On the testing day, following normal sleep or acute sleep deprivation, mice were tested for SA without sucrose pellet delivery for ~30 min followed by regular SA with pellet delivery for another ~30 min. **B** *Top*, Representative heat maps of NAc Ca^2+^ Z-scores in an example mouse across the four conditions. Each row represented an individual trial with ALP aligned at t = 0 s. *Bottom*, Averaged NAc Ca^2+^ Z-scores from all the mice during SA following normal sleep or acute sleep deprivation, in the absence or presence of sucrose pellet delivery. *Yellow shades*, periods used for AUC calculations. *Green arrow*, time of ALP; *red arrow*, time of sucrose pellet reward delivery. **C** Total AUC of NAc Ca^2+^ Z-score (−5–10 s) following normal sleep or sleep deprivation, and in the absence or presence of sucrose pellet deliveries. **D** #ALP under normal sleep or sleep deprivation in the absence or presence of sucrose pellet delivery. ALP active lever press, AUC area-under-curve, NAc nucleus accumbens, SA self-administration, SD sleep deprivation. Data were represented as mean ± SEM. Each circle represents a mouse. n = 10–11 mice in each condition. Main effect * *p* < 0.05, ** *p* < 0.01, *post-hoc* test # *p* < 0.05.

How may NAc activities adapt to changes in reward availability? We used a two-step design for the following sucrose SA test (Fig. 4A). During step-I, a 30-min test, ALP did not result in sucrose pellet delivery. Then, step-II followed for another 30 min, during which ALP resulted in sucrose pellet delivery as usual. Under normal sleep, NAc Ca^2+^ response was minimum during step-I when sucrose reward was not available, and was largely revealed during step-II; however, after acute SD, this response update based on reward availability was diminished (sleep x reward: *F*_(1, 9)_ = 5.591, *p* < 0.05, 2-way RM ANOVA; Fig. 4B, C). The behaviors showed an increase in #ALP following SD (SD x reward: *F*_(1, 9)_ = 2.375, *p* = 0.158, main effect of SD, *p* < 0.05, main effect of reward, *p* < 0.01; RM 2-way ANOVA; Fig. 4D). Together, these results suggest that the NAc Ca^2+^ activities are sensitive to reward update, which is impaired by acute SD.

## Discussion

The NAc serves as a limbic-motor interphase that mediates reward evaluation and processing [3–5]. Here, we show that the NAc Ca^2+^ dynamics during reward seeking are related to behavioral outcome and evolve through training stages. Moreover, acute SD reduces overall NAc responsiveness during reward seeking and dampens NAc sensitivity to reward update.

Reward seeking-associated NAc Ca^2+^ dynamics evolves along the course of behavioral training. As the mice acquired sucrose SA, the NAc Ca^2+^ activities started to increase especially before ALP (Fig. 2B, C). The increase in Ca^2+^ and its timing are both consistent with a recent study using Ca^2+^ imaging in a similar reward-seeking model in mice [56]. Over extended training, however, NAc Ca^2+^ activities were diminished, both before and after ALP (Fig. 2B, C), even though #ALP remained high (Fig. 2E). This suggests that the NAc is less engaged once reward-seeking behavior is proficient. Considering that fiber photometry depicts population activities, it could either be that NAc processing becomes exceedingly efficient, requiring reduced number of neurons and less overall activities to achieve similar behavioral outcomes; or that NAc processing may be largely by-passed by other regulatory circuits, for example, those that mediate habitual behaviors [57]. Indeed, at stage iv, most mice (4 out of 5) perseveringly pressed the inactive lever more than the active lever (ALP% <45%) long after the two levers were reversed – for as long as we tested, up to 7 days. This suggests that as the behavior transitions to presumed habitual, NAc activities could be less involved. Either scenario could account for the immediate increase in NAc Ca^2+^ activities when the active and inactive levers were reversed (Fig. 2B, D), which likely reengages NAc to evaluate the reward associated with the newly assigned active lever.

The NAc Ca^2+^ dynamics can be roughly divided into pre- and post-lever press responses. Whereas the post-lever press responses reflect reward delivery (Fig. 4B, S1A), how may pre-lever press responses be interpreted? It is unlikely that the NAc pre-lever press Ca^2+^ reflects motor activities, based on the following: 1) during initial training, the Non-retrievers pressed the active lever more than the Retrievers, yet their pre-ALP Ca^2+^ was low; 2) similarly, in stage iii training, pre-ALP Ca^2+^ was reduced compared to stage ii (Fig. 2C), even though #ALP remained robust (Fig. 2E); 3) under no-reward testing phase following sleep deprivation, pre-ALP Ca^2+^ was low (Fig. 4B*right*) despite augmented #ALP (Fig. 4D). Together, these results argue against the association of pre-ALP Ca^2+^ with the motor activities for lever press. On the other hand, our results are consistent with the notion that the pre-ALP activities reflect anticipation of reward that precedes self-initiated reward seeking [56, 58]. This component was increased from the beginning stage i to the learned stage ii (Fig. 2B, C), consistent with the presumed augmentation of reward anticipation as the training progressed. The pre-ALP Ca^2+^ may be also associated with the internal evaluation of the anticipated reward, as it is higher in the Retriever mice during the initial training than in Non-retrievers (Fig. 1D). Moreover, after prolonged training in stage iii the pre-ALP Ca^2+^ was decreased compared to stage ii (Fig. 2C), which could be consistent with a decrease in the salience of the anticipated reward. Subsequent reversal of levers in stage iv did not increase the pre-ALP Ca^2+^(Fig. 2D), as the anticipation of reward to be associated with a new lever pressing was yet to be established. On the other hand, stage iv pre-ILP Ca^2+^ was larger than stage i pre-ALP Ca^2+^, even though the motor aspects were similar (Fig. S1A, B). This may be accounted for by the better-established anticipation of reward in stage iv than in stage i, even if it was a false expectation as the levers were swapped. Together, the NAc pre-ALP Ca^2+^ may reflect anticipation of reward, rather than merely the motor activities leading to lever press.

Our current study does not differentiate between NAc principal neurons that predominantly express D1 type dopamine receptors (D1 neurons) versus those that express D2 dopamine receptors (D2 neurons) [59]. Using D1-Cre and D2-Cre mice, a recent study monitored NAc D1 and D2 neuron Ca^2+^ activities during self-initiated reward seeking [56]. It was shown that D1 and D2 neuron Ca^2+^ activities rise prior to lever pressing for sucrose, and moves into synchrony following initial learning, both are consistent with our observations (Fig. 2B). It was also shown that D1 neuron activities typically precede D2 neuron activities before lever pressing [56]. Regarding the changes in NAc dynamics following training, another study by Deseyve et al. measured D1 and D2 neuron responses during a Pavlovian learning paradigm in mice, and showed that both D1 and D2 neurons reduced Ca^2+^ responses to reward-associated cues at late training [13]. It remains to be determined for self-initiated reward seeking, whether the training-induced decrease in NAc Ca^2+^ responses involves both D1 and D2 neuron response changes.

Following acute SD, #ALP for sucrose was increased (Fig. 3D), whereas NAc Ca^2+^ responses were decreased (Fig. 3A–C). This is consistent with our previous reports that acute SD increases sucrose SA in mice [36, 54], and that the overall NAc activity measured by c-Fos expression is inversely correlated with #ALP for sucrose reward seeking (study under review). A tentative interpretation is that the NAc activities reflect the level of arousal, consistent with the accumulating evidence that NAc diverse cell types and projections regulate sleep and wakefulness [60–65], which is intimately associated with reward seeking. Thus, reduced arousal – such as that following SD – may bias toward habitual behaviors. Similarly, during prolonged SA training (Fig. 2B, C), arousal may also be suboptimal. By contrast, during stage iv reversal training which likely involves heightened arousal, NAc Ca^2+^ response was elevated (Fig. 2B, D). Nonetheless, following normal sleep under no-reward condition, NAc Ca^2+^ response was minimal, even though the mice appeared to be highly aroused – #ALP was higher than under reward condition (Fig. 4D). Thus, NAc Ca^2+^ response may be intersectionally associated with arousal and reward, requiring both for full activation. Along this reasoning, high arousal combined with reward may fully engage NAc activities for adaptive learning and decision-making processes [24, 66, 67], which may be compromised under SD.

Regarding the cellular mechanisms underlying SD-induced dampening of NAc responses, one possibility is that SD reduces the excitatory synaptic inputs onto NAc neurons. For example, both prefrontal cortical and rostral amygdala glutamatergic inputs to NAc shell principal neurons show reduced glutamate release [36, 54], and the overall excitatory/inhibitory balance in synaptic transmission onto these neurons is shifted toward inhibition following acute SD [36, 54]. Likewise, a human imaging study demonstrated that poor sleep decreased the coupling between the dorsal lateral PFC and NAc during reward processing suggesting altered top-down regulation [68]. Thus, reduced excitatory drives may decrease NAc activities during reward seeking. At the behavioral level, this may render the NAc less responsive to reward, which in turn, precipitates habitual behaviors. Our results are in contrast to human imaging studies which predominantly document an SD-induced increase in NAc response to reward or reward-associated cues, which accompanies increased motivation for reward [28–32, 53]. There are a few potential differences, including that, *i*) we monitored self-initiated reward seeking rather than cue-elicited reward seeking; *ii*) we used well-trained mice that took the task as a daily routine rather than associating the task with a highly salient reward such as money – often used in human studies; and *iii*) our mice had free access to food during SD, whereas human studies on food reward typically require a fasting period before the test [28, 29, 53]. Thus, there could be distinct neural mechanisms that contribute to SD-induced increase in natural reward seeking under different circumstances.

SD not only reduced NAc Ca^2+^ activities during learned reward-seeking behavior (Fig. 3), but also blunted its response to changes in reward availability (Fig. 4B, C). This is consistent with the notion that SD promotes habitual over goal-directed reward-seeking behaviors [69], and impairs behavioral flexibility specifically through “feedback blunting” [70]. The underlying neural mechanisms are not clear, though possibly related to the decreased functional coupling between the PFC and subcortical regions, in the context of reducing top-down dynamic attentional controls [70].

## Supplementary information

Supplementary material

## Data Availability

All data needed to evaluate the conclusions in the paper are present in the paper or the Supplementary Materials.

## Code availability

All MATLAB scripts were based on version R2022a and are available upon request.
