# Supplementary material for: Nucleus accumbens dynamics in food reward seeking and regulation by sleep
Source: Transl Psychiatry. 2025 Jul 1;15:219. doi: 10.1038/s41398-025-03442-z (PMC12218339; doi:10.1038/s41398-025-03442-z)
Supplement: Supplementary file 1 — Supplementary material [file 41398_2025_3442_MOESM1_ESM.pdf]

Supplementary Materials for

Nucleus Accumbens Dynamics in Food Reward Seeking and Regulation by Sleep

Almeida Rojo *et al.*

*Corresponding author. Email: yhhuang@pitt.edu

This PDF file includes:

Fig. S1

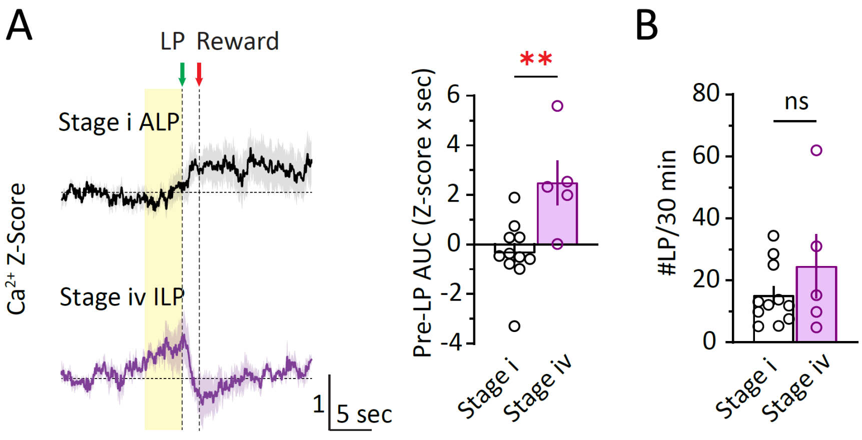

Fig. S1 NAc Ca^{2+} responses to inactive-lever press during stage iv reversal training. **A** *Left* Group-averaged Z-scores of NAc Ca^{2+} in response to ILP in stage iv reversal training aligned with stage i Ca^{2+} to ALP, shown as Mean +/- SEM from all mice in each group. *Right* Grouped data showing larger pre-lever press Ca^{2+} in stage iv compared to that in stage i, calculated as AUC from -5 sec till 0 sec. t_{14}=3.456, $p < 0.01$; *t*-test. **B** Similar #lever press (i.e. stage i #ALP versus stage iv #ILP) in the two groups as in **A**. t_{14}=1.175, $p = 0.260$; *t*-test. Each circle represents a mouse. ALP, active lever press; AUC, area-under-curve; ILP, inactive lever press, LP, lever press. Data were represented as mean ± SEM. n = 5 – 11 mice in each group. ** $p < 0.01$, ns = not significant.
